# Age-Specific Differences in Laryngotracheal Trauma Characteristics: A Retrospective Study of Clinical Profiles, Outcomes, and Mortality Risk

**DOI:** 10.3390/jcm13123508

**Published:** 2024-06-15

**Authors:** Ahmad K. Alnemare

**Affiliations:** Department of Otolaryngology, Faculty of Medicine, Majmaah University, Al-Majmaah 11952, Saudi Arabia; a.alnemare@mu.edu.sa

**Keywords:** geriatric trauma, laryngotracheal injuries, airway compromise, emergency airway surgery

## Abstract

**Background**: Laryngotracheal trauma is associated with a substantial risk of mortality. Age can be a critical factor in trauma management, as older adults often have diminished airway protective reflexes and preexisting respiratory conditions. Objective: This study aimed to characterize the clinical profiles and outcomes in different age groups of adult patients with laryngotracheal trauma using data from the National Trauma Data Bank (NTDB). **Methods**: We retrospectively analyzed the NTDB and included adult patients (aged ≥ 18 years) who had laryngotracheal fractures (closed or open) and were admitted directly after the injury. The patients were categorized into different age groups for analysis. A multivariate logistic regression analysis was performed to assess whether the elderly population (age ≥ 65 years) was predisposed to post-trauma death under care. **Results**: The study included 1171 patients, with the following age distributions: 13.7% aged 18–24 years, 21.6% aged 25–34 years, 55.2% aged 35–64 years, and 9.6% aged ≥ 65 years. Notable differences were observed in comorbidities, mechanisms, types of injuries, and associated injuries among age groups. There was no significant trend in airway surgical outcomes according to age. In-hospital mortality was highest among patients aged ≥ 65 years (22.3%), compared to 14.4% for those aged 18–24 years. Regression analysis indicated that age ≥ 65 was an independent mortality predictor. **Conclusions**: These findings underscore significant age-related differences in the presentation and outcomes of laryngotracheal trauma, emphasizing the need for age-specific treatment protocols, primarily to address the elevated risk among elderly patients.

## 1. Introduction

Trauma is a major global health issue responsible for a significant number of preventable deaths across all age groups [1]. Laryngotracheal trauma refers to injuries to the larynx and trachea that can have high morbidity and mortality rates if not managed appropriately [2,3,4]. It is an emerging high-risk condition that disproportionately affects vulnerable subgroups. Injuries to the larynx, trachea, cricoid, or supporting structures carry substantial early mortality, approaching 35% in some reports, due to the threats of asphyxiation and hemorrhage [5,6]. Blunt mechanisms such as motor vehicle collisions, clothesline injuries, and penetrating neck trauma contribute to airway compromise syndromes [7,8,9,10].

Managing laryngotracheal trauma involves securing the airway, controlling hemorrhage, and repairing damaged structures. In severe cases, immediate recognition and intervention are essential, with general guidelines advocating emergency tracheostomy or cricothyroidotomy to establish airway security and allow for operative exploration [11,12]. Conservative approaches may be selected based on the severity and location of the injury. Timely intervention is essential to prevent complications such as shock, sepsis, and laryngeal stenosis. Postoperative care, including vocal rest, speech therapy, and swallowing therapy, may be necessary for a successful recovery. However, current data on the efficacy of various surgical strategies remains limited, especially among elderly patients with comorbid conditions that complicate risk assessment [13].

Factors such as hemorrhage-induced shock, age, and comorbidities can predict the risk of mortality in severely injured patients. However, trauma management in developing countries faces challenges such as inadequate prehospital care and limited resources [14]. The global incidence of trauma is expected to increase, requiring a focus on prevention and public health [15]. Age extremes have emerged as uniquely vulnerable populations in the context of injuries. Patients with geriatric trauma, typically defined as those older than 65 years [16], experience disproportionately worse outcomes, complications, length of stay, discharge disposition, readmissions, and mortality [17,18]. Trauma essentially represents an accelerated model of frailty in seniors, with reduced physiological reserves and comorbid conditions that inhibit the ability to compensate for acute stress [19,20].

Outcomes lag even after adjusting for injury severity, given age-related differences in immune function, medication metabolism, nutrition, cognition, and post-discharge support. Trauma manifests itself as a particularly morbid disease in extremes of age, from young to middle-aged adults [20,21,22]. Youth violence prevention programs require detailed neighborhood-level data to target specific community needs based on demographically specific trauma etiologies. As the US undergoes rapid aging, with increasing numbers of seniors surviving in their late 70s, 80s, and beyond, it is critical to clarify the patterns of geriatric injuries and the efficacy of associated interventions [21,22].

Therefore, in this study, our objective was to utilize the NTDB database in geriatric and younger adult patients with blunt or penetrating laryngotracheal trauma [23,24]. We hypothesized that early geriatric (age ≥ 65 years) [16] laryngotracheal trauma victims are associated with higher hospital mortality and different trauma mechanisms and injury characteristics than younger trauma victims (18–64 years). As a secondary goal, we sought to characterize the frequency of significant airway surgeries in this cohort and the effect of trauma outcomes in terms of mortality and complications. Clarifying the evidence base for high-risk airway interventions in this vulnerable trauma subgroup is necessary to inform clinical decision-making algorithms and protocols directly to allow patient-centered advancements in geriatric trauma care equity.

## 2. Methods

### 2.1. Study Design and Data Source

This retrospective study used data from the National Trauma Data Bank (NTDB) from January 2013 to December 2015.

### 2.2. Inclusion and Exclusion Criteria

The study included adult patients (18 years and older) with laryngotracheal trauma diagnosed with a closed larynx and tracheal fracture (ICD-9-CM code 807.5) or an open larynx and tracheal fracture (ICD-9-CM code 807.6). Both sexes were included. Minor airway surgeries include procedures that cover interventions, such as sutures of the larynx and temporary tracheostomies. Major airway surgeries include extensive procedures such as laryngectomy and permanent tracheostomy. Eligible participants were those admitted directly to the site of the injury. We excluded patients transferred from other facilities to avoid complications related to prior treatments, which could have skewed the outcomes studied. Additionally, patients who died upon arrival were excluded to focus on those who received active trauma care, thus providing a clearer picture of treatment efficacy and outcomes.

### 2.3. Variables and Outcomes

Data on demographic variables, mechanisms of injury, injury severity scores, vital signs at admission, comorbidities, length of hospital stay, and in-hospital mortality were extracted. The primary outcomes were complications, intensive care unit admission, and in-hospital mortality. The demographic variables included age, sex, and race. Age was categorized into four groups: 18–24 years, 25–44 years, 45–64 years, and ≥65 years to allow for age-specific analyses. Complications were identified using ICD-9-CM diagnosis codes, including cardiovascular and other complications. ICU admission was determined based on the presence of ICU stay during hospitalization. In-hospital mortality was defined as death from any cause during the hospital stay.

### 2.4. Statistical Analysis

The dataset was stratified as 18–24 years, 25–44 years, 45–64 years, and ≥65 years. Descriptive statistics were used to characterize demographics, injury patterns, resource utilization, and unadjusted outcomes between groups using the Kruskal–Wallis and chi-square tests. Multivariate logistic regression models were used to identify the independent predictors of mortality after controlling for confounders. Missing data were addressed through listwise deletion. Statistical analysis was performed using Stata 15.1, with significance defined as *p* < 0.05.

## 3. Results

### 3.1. Demographic Characteristics

The analysis included 1171 patients with laryngotracheal trauma (closed, 83.9%; open, 16.1%): 160 (13.7%) were aged 18–24 years, 253 (21.6%) were aged 25–34 years, 646 (55.2%) were aged 35–64 years, and 112 (9.6%) were aged ≥ 65 years. Data for different age groups are presented in Table 1, reflecting the significant differences in demographics and injury characteristics. In particular, the median age was 22 years for 18–24-year-olds and 72 years for 112 (100%) in the 65+ group (*p* < 0.001). Racial differences existed, as 47 (29.4%) of the 18–24-year-olds were Black compared to 16 (14.3%) of the 112 patients aged ≥ 65 years (*p* = 0.045).

### 3.2. Trauma Characteristics

Significant differences were observed in the mechanism of injury across the age groups (*p* < 0.001, Table 1). Fall injuries increased with age, affecting 26.8% of those aged 65+ years compared to just 1.3% of 18–24 year olds. However, firearm injuries showed the opposite pattern, accounting for 28.1% of injuries among 18–24-year-olds versus 6.3% for those aged 65 years and older. Motor vehicle trauma (MVT) was common in all the groups. Age-based differences in trauma types were also significant (*p* < 0.001). Blunt trauma was the most common overall, although 18–24 year-olds had higher rates of penetrating trauma (32.1%) than patients aged ≥ 65 years (12.6%). Burns and other/unspecified trauma types were rare in all age groups. The differences in injury intention approached significance according to age (*p* = 0.054). Assault accounted for a higher proportion of injuries among 18–24-year-olds (35.2%) than among those aged 65+ (18.9%). In contrast, unintentional trauma was more common in 65+ patients (71.2% vs. 56.0% for ages 18–24).

### 3.3. Comorbidities

The prevalence of substance abuse was significantly higher in the 25–34 (15.0%) and 35–64 (13.9%) age groups than in the 18–24 (8.8%) and 65 + (1.8%) age groups (*p* = 0.001, Table 2). Cardiovascular health issues were more prevalent in older patients, particularly in those aged ≥ 65 years (3.6%), which was significantly different from that in the younger age groups (*p* = 0.012). Major psychiatric illness, or dementia, also showed an increasing trend with age, being highest in the 65+ group (12.5%) and lowest in the 18–24 group (6.9%), although this difference was not statistically significant (*p* = 0.425). Chronic health conditions were significantly more prevalent in the older age groups, 21.4% in the 65+ group compared to only 0.6% in the 18–24 group (*p* < 0.001). Blood disorders were also more common in older patients, particularly in those aged ≥ 65 years (5.4%) than in younger patients (*p* = 0.001). Other primary health conditions followed a similar pattern, being more prevalent in the ≥ 65 years age group (4.5%) than in the younger age groups (*p* < 0.001).

### 3.4. Use of Protective Devices

Regarding protective equipment, airbags and helmets did not vary significantly across the age groups (Table 2). However, there was a notable trend in the non-use of protective devices, with the highest non-use reported in the 25–34 age group (51.4%) and the lowest in the 18–24 age group (38.1%), although this difference was not statistically significant (*p* = 0.060).

### 3.5. Concomitant Injuries

In terms of concomitant injuries, intracranial injuries were more common in the 18–24 (30.6%) and 35–64 (28.5%) age groups than in the 25–34 (25.3%) and 65+ (26.8%) age groups; however, this difference was not statistically significant (*p* = 0.655, Table 2). Injury to blood vessels was most prevalent in the 25–34 age group (19.4%) and least prevalent in the 65+ years group (10.7%), with a significant difference across age groups (*p* = 0.018). The incidence of nerve or spinal cord injuries was relatively low across all age groups, with no significant differences observed (*p* = 0.157).

### 3.6. Vital Signs

We found significant variations in vital parameters in the analysis of clinical characteristics and treatment outcomes across different age groups among the 1171 trauma patients. As described in Table 3, there was a noticeable increase in median systolic blood pressure (SBP) with age, ranging from 120.5 mmHg in the 18–24 group to 134.0 mmHg in the 65+ group (*p* < 0.001). Pulse rate (PR) and respiratory rate (RR) did not show significant age-related differences. However, Glasgow Coma Scale (GCS) scores indicated that younger patients (18–24 and 25–34 age groups) had lower median scores than the older groups, with significant differences observed (*p* = 0.336).

Regarding the state of the patients, 45.6% of those aged 18–24 were comatose compared to 37.5% in the 65+ age group, but these differences were not statistically significant (*p* = 0.091). The proportion of severely injured patients was highest in the 18–24 age group (53.1%) and lowest in the 25–34 age group (44.3%); however, the differences were not significant (*p* = 0.335). Hemodynamic instability was highest in the 18–24 age group (31.9%) and lowest in the 65+ years group (22.3%), without a significant age-related trend (*p* = 0.211).

### 3.7. Major Airway Procedures and Outcomes

Regarding surgical interventions, the percentage of patients undergoing major airway surgery decreased with age, from 29.4% in the 18–24 group to 21.8% in the 65+ group, although this was not statistically significant (*p* = 0.201, Table 3). Minor airway surgeries and surgeries of the nervous and endocrine systems do not show a clear age-related pattern. Surprisingly, surgeries on the respiratory system were more common in younger patients, particularly in the 18–24 and 25–34 age groups, than in older patients. However, these differences were not statistically significant (*p* = 0.111).

Complications after trauma varied slightly between the age groups. Respiratory and cardiovascular complications increased slightly in the older age groups, but the differences were insignificant (*p* = 0.736 and *p* = 0.670, respectively). Infection complications were more common in the 65+ group (5.4%) than in the younger group; however, these differences were not statistically significant (*p* = 0.262). Surgical complications and intracranial hemorrhage did not show any significant age-related trends.

In general, the prevalence of death under care (DUC) was highest in the 65+ age group (22.3%) and lowest in the 18–24 age group (14.4%), but these differences were not statistically significant (*p* = 0.233). These findings suggest age-related differences in the clinical characteristics and outcomes, underscoring the importance of age-specific considerations in the management of patients with trauma.

### 3.8. Multivariate Logistic Regression for Mortality Predictors

In the analysis of factors contributing to DUC among trauma patients, key predictors (Table 4) were identified, displaying odds ratios (OR), 95% confidence intervals (CI), and *p*-values. Systolic blood pressure (SBP) had no significant effect on mortality (OR = 1.00, *p* = 0.980). In contrast, the injury severity score adjusted for injury severity (ISSAIS) significantly predicted higher mortality (OR = 1.09, *p* < 0.001). The total Glasgow Coma Scale score (GCSTOT) indicated a significant effect (OR = 0.87, *p* < 0.001). Age ≥ 65 years notably increased mortality risk (OR = 2.60, *p* = 0.003). The logistic regression model demonstrated a predictive solid performance, with an area under the ROC curve of 0.88, indicating good model accuracy in distinguishing between the outcomes.

## 4. Discussion

This study analyzed the clinical characteristics and outcomes of 1171 patients with trauma and uncovered significant age-related variations in injury mechanisms, comorbidities, and treatment outcomes. Our findings offer critical insights into the differing patterns of trauma across various age groups, highlighting the complex interplay between demographic factors and the severity of trauma. In particular, this study revealed different trends in the prevalence of specific types of injuries, such as fall-related trauma in older adults and firearm injuries in younger patients. In this analysis of more than 1100 adult trauma patients, we sought to characterize the injury patterns, resource utilization, complications, and mortality outcomes in different age groups. We found significant age-based differences in pre-existing comorbidities, injury severity, length of hospital stay, complications, and death rates. Specifically, geriatric patients (≥65 years) had worse outcomes than their younger (18–64 years) counterparts across nearly all metrics: they had higher injury severity, almost 50% longer hospital stays, elevated rates of complications such as pneumonia and sepsis, and a significantly increased risk of dying under care, even after adjusting for trauma burden. Our findings reinforce geriatric trauma as a distinct clinical entity that requires tailored management strategies to address the unique needs of elderly patients.

The higher prevalence of fall injuries and blunt trauma among older patients, as revealed in our study, raises significant concerns regarding the vulnerability of this demographic [25]. Falls in the elderly often indicate underlying frailty and can have devastating consequences, including prolonged hospitalization and increased mortality [26,27]. This trend suggests a critical need for enhanced fall prevention strategies, such as community-based programs focusing on balance and strength training, safer home environments, and comprehensive geriatric assessments, to identify risk factors. In contrast, the increased incidence of firearm injuries in younger patients, particularly those aged 18–24 years, highlights a different spectrum of trauma etiology. This finding may reflect social and environmental factors, such as exposure to violence or high-risk behaviors [28,29,30,31]. The prominence of firearm injuries in this age group underscores the need for targeted violence prevention initiatives, including community outreach programs, education on firearm safety, and policy measures aimed at reducing gun violence. These distinct patterns of trauma between age groups require a dual approach to trauma care and prevention. For older adults, healthcare systems should focus on fall prevention, the early detection of frailty, and customized rehabilitation programs. For younger populations, emphasis should be placed on injury prevention strategies that address high-risk behaviors and societal factors that contribute to violent injuries. Understanding these age-specific trends is crucial for policymakers and healthcare providers to develop effective targeted interventions that address the unique needs of each population group, ultimately improving the results and reducing the burden of trauma. The high prevalence of low-speed falls as a mechanism of injury in patients over 65 years of age (48.2%), coupled with exceedingly high rates of blunt trauma (96.4%), has significant implications for preventive initiatives and trauma practices. Fall risks, such as home hazards and the side effects of medications, should be routinely assessed in older adults. Trauma centers would benefit from geriatric-specific guidelines that address the nuanced clinical profile introduced by ground-level falls compared to higher-velocity mechanisms. Among younger patients aged 18–24 years, penetration mechanisms, including firearms and knives, accounted for over 50% of trauma cases, exceeding those in the older groups. This highlights the need for tailored preventive strategies focused on conflict mediation, which means there are restrictions on this demographic. Younger patients were also significantly more likely to arrive uninsured, an obstacle that must be addressed to facilitate access to risk counseling and violence interruption programs.

In our study, SBP, a commonly monitored vital sign in trauma care, did not emerge as a significant predictor of DUC [32]. This suggests that, while SBP is crucial for immediate physiological assessment, its role in long-term outcomes, particularly in diverse trauma populations, may be limited. This insight calls for a broader approach to the evaluation of trauma patients, in which SBP is considered in conjunction with a variety of other clinical indicators. In contrast, the ISS and total GCS score were significant predictors of DUC. The ISS, which quantifies the overall severity of injuries, is crucial for determining patient outcomes. This underscores the importance of a comprehensive injury assessment to guide treatment decisions and prognosis. Similarly, GCSTOT, as a measure of consciousness level and neurological function, proved crucial in predicting patient outcomes, reinforcing its value in the initial assessment and ongoing monitoring of patients with trauma. Furthermore, the impact of chronic health conditions and psychiatric diseases on trauma outcomes was evident. These comorbidities can complicate patient management, affect recovery trajectories, and potentially worsen patient outcomes. For instance, pre-existing cardiovascular conditions or mental health issues can influence both the response to trauma and the recovery process. These findings emphasize the need for holistic patient evaluation in trauma care, taking into account not only the immediate injury but also the broader context of the patient’s overall health and pre-existing conditions. Integrating this comprehensive approach could lead to more individualized and effective care strategies, ultimately improving outcomes for patients with trauma. It is important to consider the potential interaction between comorbidities and injury outcomes in this population. Elderly patients often present with a higher burden of comorbidities such as cardiovascular disease, respiratory disorders, and diabetes, which may complicate their recovery from laryngotracheal trauma. The presence of these comorbidities may interact with injury severity and other clinical factors, leading to worse outcomes. For example, patients with pre-existing respiratory conditions may be more susceptible to respiratory complications following laryngotracheal trauma, which could contribute to increased mortality risk. Similarly, cardiovascular comorbidities may limit the ability of elderly patients to compensate for the physiological stress associated with injury and its management.

Blunt mechanisms collectively account for more than 75% of traumas in this population. However, after adjustment, only low-velocity injuries from being struck by or against objects significantly reduced the mortality risk compared with penetrating trauma. This suggests that injury kinetics and energy transfer dictate severity rather than broad categories of mechanisms. Tailored prevention initiatives should target demographic-specific patterns, as age extremes demonstrate divergent trauma profiles. Gender-based differences in risk-taking behaviors and alcohol use can also influence violent outreach among youth. Trauma guidelines must also evolve to address injury patterns from emerging mechanisms based on lifestyle and technological trends. The mechanism of injury plays a crucial role in determining the likelihood of a DUC.

### 4.1. Clinical and Policy Implications

The findings of this study have significant implications for clinical practice and public health policy. Clinically, they underscore the need for age-specific trauma care protocols, emphasizing the importance of rapid airway management and tailored surgical intervention. From a policy perspective, the data advocate targeted public health initiatives, such as violence prevention programs for younger people and fall prevention strategies for the elderly. Enhancing community awareness and education regarding risks and preventive measures for specific types of trauma can also be beneficial.

### 4.2. Limitations and Future Research

The limitations of our study stem primarily from its retrospective design and the nature of the data sources used. The NTDB, while extensive, is subject to selection bias due to the voluntary participation of hospitals. This means that hospitals that choose to participate may not be representative of all trauma centers, potentially skewing our findings and limiting their generalizability. Furthermore, retrospective analysis limits our ability to establish causality between observed factors and outcomes, as it inherently depends on the accuracy and completeness of the recorded data. Additionally, the exclusion of variables such as socioeconomic factors and long-term outcomes from the NTDB dataset further constrained our conclusions. These omitted variables are crucial for understanding the full impact of trauma and the effectiveness of interventions across different populations. To address these limitations, future research should consider a more inclusive dataset and possibly a prospective study design to better control and identify causal relationships. It is also important to incorporate a wider range of demographic and socioeconomic variables to enhance the applicability of these findings across diverse patient groups.

## 5. Conclusions

This study demonstrated significant age-related differences in injury patterns, resource utilization, complications, and mortality among adult patients with trauma. Geriatric patients have worse outcomes than their younger counterparts; they have a higher severity of injuries, more extended hospital stays, more complications, and a nearly two-fold more significant risk of dying, even after adjusting for trauma burden. These findings categorize geriatric trauma as a distinct clinical entity compared with younger or middle-aged adults. Trauma guidelines and practices must adapt to the needs of an aging population at an increased risk of ground-level falls and associated head strikes. In parallel, aggressive injury prevention approaches should target youth violence to address the demographic patterns. We can only address the care gaps illuminated by this analysis through age-specific interventions, both public health initiatives and specialized clinical pathways. Additional research exploring long-term functional recovery and patient-centered outcomes will advance trauma care equity throughout life.

## Figures and Tables

**Table 1:** Demographic and clinical characteristics of patients by age group.

Variable	Age Group				
	18–24 (*N* = 160)	25–34 (*N* = 253)	35–64 (*N* = 646)	65+ (*N* = 112)	*p*-Value
Age (Median, Q1, Q3)	22.0 (20.0, 23.0)	29.0 (27.0, 32.0)	48.0 (42.0, 55.0)	72.0 (68.0, 79.5)	<0.001
Gender-Male (%)	141 (88.1%)	221 (87.4%)	560 (86.7%)	89 (79.5%)	0.412
Race Category (%)					0.045
Black	47 (29.4%)	58 (22.9%)	140 (21.7%)	16 (14.3%)	
Others	28 (17.5%)	56 (22.1%)	120 (18.6%)	18 (16.1%)	
White	85 (53.1%)	139 (54.9%)	386 (59.8%)	78 (69.6%)	
Mechanism of Injury (%)					<0.001
Cut/Pierce	6 (3.8%)	12 (4.7%)	39 (6.0%)	7 (6.3%)	
Fall	2 (1.3%)	12 (4.7%)	54 (8.4%)	30 (26.8%)	
Firearm	45 (28.1%)	51 (20.2%)	56 (8.7%)	7 (6.3%)	
MVT	60 (37.5%)	76 (30.0%)	197 (30.5%)	30 (26.8%)	
Other	23 (14.4%)	45 (17.8%)	126 (19.5%)	19 (17.0%)	
Pedal/Pedestrian	3 (1.9%)	4 (1.6%)	29 (4.5%)	4 (3.6%)	
Struck By/Against	21 (13.1%)	43 (17.0%)	130 (20.1%)	12 (10.7%)	
Unspecified	0 (0.0%)	10 (4.0%)	15 (2.3%)	3 (2.7%)	
Intention of Injury (%)					0.054
Assault	56 (35.2%)	97 (38.3%)	201 (31.5%)	21 (18.9%)	
Other	1 (0.6%)	2 (0.8%)	5 (0.8%)	0 (0.0%)	
Self-inflicted	13 (8.2%)	29 (11.5%)	56 (8.8%)	10 (9.0%)	
Undetermined	0 (0.0%)	1 (0.4%)	5 (0.8%)	1 (0.9%)	
Unintentional	89 (56.0%)	124 (49.0%)	372 (58.2%)	79 (71.2%)	
Trauma Type (%)					<0.001
Blunt	101 (63.5%)	147 (58.1%)	459 (71.8%)	85 (76.6%)	
Burn	0 (0.0%)	0 (0.0%)	1 (0.2%)	0 (0.0%)	
Other/Unspecified	7 (4.4%)	43 (17.0%)	84 (13.1%)	12 (10.8%)	
Penetrating	51 (32.1%)	63 (24.9%)	95 (14.9%)	14 (12.6%)	

**Table 2:** Prevalence of health conditions and use of protective devices by age group.

Variable	Age Group				
	18–24 (*N* = 160)	25–34 (*N* = 253)	35–64 (*N* = 646)	65+ (*N* = 112)	*p*-Value
**Comorbidities**					
*Substance Abuse Presence (%)*	14 (8.8%)	38 (15.0%)	90 (13.9%)	2 (1.8%)	0.001
*Cardiovascular Health Issues (%)*	0 (0.0%)	0 (0.0%)	10 (1.5%)	4 (3.6%)	0.012
*Major Psychiatric Illness or Dementia (%)*	11 (6.9%)	24 (9.5%)	68 (10.5%)	14 (12.5%)	0.425
*Chronic Health Conditions (%)*	1 (0.6%)	3 (1.2%)	50 (7.7%)	24 (21.4%)	<0.001
*Blood Disorders Presence (%)*	0 (0.0%)	1 (0.4%)	9 (1.4%)	6 (5.4%)	0.001
*Other Major Health Conditions (%)*	0 (0.0%)	0 (0.0%)	4 (0.6%)	5 (4.5%)	<0.001
**Protective Equipments**					
*Airbag Present (%)*	34 (21.3%)	45 (17.8%)	113 (17.5%)	17 (15.2%)	0.601
*Helmet (%)*	16 (10.0%)	27 (10.7%)	80 (12.4%)	11 (9.8%)	0.722
*Lap/Shoulder Belts (%)*	17 (10.6%)	15 (5.9%)	42 (6.5%)	10 (8.9%)	0.221
*No Protective Device (%)*	61 (38.1%)	130 (51.4%)	311 (48.1%)	51 (45.5%)	0.060
*Airbag Deployed Front (%)*	10 (6.3%)	14 (5.5%)	22 (3.4%)	5 (4.5%)	0.305
*Airbag Not Deployed (%)*	2 (1.3%)	0 (0.0%)	5 (0.8%)	2 (1.8%)	0.265
**Associated injuries**					
*Intracranial Injury*	49 (30.6%)	64 (25.3%)	184 (28.5%)	30 (26.8%)	0.655
*Injury to Blood Vessels*	27 (16.9%)	49 (19.4%)	78 (12.1%)	12 (10.7%)	0.018
*Nerve or Spinal Cord Injury*	10 (6.3%)	6 (2.4%)	20 (3.1%)	3 (2.7%)	0.157

**Table 3:** Clinical characteristics and treatment outcomes by age group.

Variable	Age Group				
	18–24 (*N* = 160)	25–34 (*N* = 253)	35–64 (*N* = 646)	65+ (*N* = 112)	*p*-Value
**Vital parameters**					
*SBP (mmHg) [Median, Q1, Q3]*	120.5 (0.0, 140.0)	124.0 (86.0, 140.0)	128.0 (90.0, 150.0)	134.0 (100.0, 162.0)	<0.001
*PR (bpm) [Median, Q1, Q3]*	88.5 (7.0, 103.0)	90.0 (66.0, 106.0)	89.0 (66.0, 104.0)	85.0 (68.5, 102.0)	0.429
*RR (breaths/min) [Median, Q1, Q3]*	16.0 (0.0, 20.0)	16.0 (1.0, 20.0)	16.0 (8.0, 20.0)	17.0 (10.5, 20.0)	0.787
*GCS [Median, Q1, Q3]*	12.0 (3.0, 15.0)	11.0 (3.0, 15.0)	14.0 (3.0, 15.0)	14.0 (3.0, 15.0)	0.336
*Comatose (%)*	73 (45.6%)	118 (46.6%)	251 (38.9%)	42 (37.5%)	0.091
*Severely Injured (%)*	85 (53.1%)	112 (44.3%)	297 (46.0%)	52 (46.4%)	0.335
*Hemodynamically Unstable (%)*	51 (31.9%)	64 (25.3%)	157 (24.3%)	25 (22.3%)	0.211
**Surgical Interventions**					
*Operative (%)*	122 (76.3%)	191 (76.7%)	476 (74.6%)	76 (69.1%)	0.463
*Major Airway Surgery (%)*	47 (29.4%)	61 (24.5%)	138 (21.6%)	24 (21.8%)	0.201
*Minor Airway Surgery (%)*	2 (1.3%)	7 (2.8%)	14 (2.2%)	1 (0.9%)	0.576
*Operations on the Nervous System (%)*	13 (8.1%)	26 (10.4%)	41 (6.4%)	9 (8.2%)	0.244
*Operations on the Endocrine System (%)*	14 (8.8%)	17 (6.8%)	34 (5.3%)	1 (0.9%)	0.042
*Operations on the Nose, Mouth, and Pharynx (%)*	24 (15.0%)	31 (12.4%)	108 (16.9%)	14 (12.7%)	0.329
*Operations on the Respiratory System (%)*	89 (55.6%)	141 (56.6%)	318 (49.8%)	50 (45.5%)	0.111
*Operations on the Cardiovascular System (%)*	50 (31.3%)	89 (35.7%)	198 (31.0%)	34 (30.9%)	0.578
**Complications**					
*Respiratory Complications (%)*	8 (5.0%)	19 (7.5%)	45 (7.0%)	9 (8.0%)	0.736
*Cardiovascular Complications (%)*	7 (4.4%)	12 (4.7%)	29 (4.5%)	8 (7.1%)	0.670
*Infection Complications (%)*	3 (1.9%)	6 (2.4%)	15 (2.3%)	6 (5.4%)	0.262
*Surgical Complications (%)*	2 (1.3%)	2 (0.8%)	7 (1.1%)	0 (0.0%)	0.698
**Intracranial Hemorrhage (%)**	1 (0.6%)	0 (0.0%)	0 (0.0%)	0 (0.0%)	0.097
**DUC (%)**	23 (14.4%)	43 (17.0%)	97 (15.0%)	25 (22.3%)	0.233

DUC, Death Under Care, GCS: Glasgow Coma Scale, PR: Pulse Rate, RR: Respiratory Rate, SBP: Systolic Blood Pressure.

**Table 4:** Odds ratios with 95% confidence intervals and p-values for predictors of death under care.

Variable	OR [95% CI]	*p*-Value
SBP	1.00 [0.996 to 1.003]	0.980
ISSAIS	1.09 [1.08 to 1.11]	<0.001
GCSTOT	0.87 [0.841 to 0.909]	<0.001
Age ≥ 65	2.60 [1.39 to 4.88]	0.003
Gender (Male vs. Female)	0.63 [0.364 to 1.093]	0.100
Injury Type: Bunt vs. Penetrating	0.89 [0.145 to 5.44]	0.898
Mechanism: Fall	0.77 [0.145 to 4.06]	0.756
Mechanism: Firearm	1.43 [0.415 to 4.91]	0.573
Mechanism: MVT	1.55 [0.358 to 6.67]	0.559
Mechanism: Other	1.28 [0.391 to 4.20]	0.682
Mechanism: Pedal/Pedestrian	1.30 [0.173 to 9.72]	0.801

SBP: Systolic Blood Pressure; ISSAIS: Injury Severity Score Adjusted for Injury Severity; GCSTOT: Total Glasgow Coma Scale score; MVT: Motor Vehicle Traffic accident.

## Data Availability

No new data were created or analyzed in this study. Data sharing is not applicable to this article.

## Abbreviations

DUC	Death Under Care
GCS	Glasgow Coma Scale
ICU	Intensive care unit
ISS	Injury Severity Score
MVT	Motor vehicle trauma
NTDB	National Trauma Data Bank
OR	Odds ratio
PR	Pulse rate
RR	Respiratory rate
SBP	Systolic blood pressure
